# Single-Cell Analysis of Circulating Tumor Cells: How Far Have We Come in the -Omics Era?

**DOI:** 10.3389/fgene.2019.00958

**Published:** 2019-10-17

**Authors:** Elisabetta Rossi, Rita Zamarchi

**Affiliations:** ^1^Department of Surgery, Oncology and Gastroenterology, University of Padova, Padova, Italy; ^2^Veneto Institute of Oncology IOV-IRCCS, Padua, Italy

**Keywords:** circulating tumor cells (CTCs), single-cell analysis, NGS, tumor heterogeneity, precision oncology

## Abstract

Tumor cells detach from the primary tumor or metastatic sites and enter the peripheral blood, often causing metastasis. These cells, named Circulating Tumor Cells (CTCs), display the same spatial and temporal heterogeneity as the primary tumor. Since CTCs are involved in tumor progression, they represent a privileged window to disclose mechanisms of metastases, while -omic analyses at the single-cell level allow dissection of the complex relationships between the tumor subpopulations and the surrounding normal tissue. However, in addition to reporting the proof of concept that we can query CTCs to reveal tumor evolution throughout the continuum of treatment for early detection of resistance to therapy, the scientific literature has also been highlighting the disadvantages of CTCs, which hampers a routine use of this approach in clinical practice. To date, an increasing number of CTC technologies, as well as -omics methods, have been employed, mostly lacking strong comparative analyses. The rarity of CTCs also represents a major challenge, because there is no consensus regarding the minimal criteria necessary and sufficient to define an event as CTC; moreover, we cannot often compare data from of one study with that of another. Finally, the availability of an individual tumor profile undermines the traditional histology-based treatment. Applying molecular data for patient benefit implies a collective effort by biologists, bioengineers, and clinicians, to create tools to interpret molecular data and manage precision medicine in every single patient. Herein, we focus on the most recent findings in CTC −omics to learn how far we have come.

## Introduction

“I know I know nothing.” (*Socrates, 470-399 B.C.*)

It is generally agreed that the birth date of the genomic era is April 14, 2003, when human genome sequencing was declared complete. If we, as scientists, think about the scenario today, 16 years later, we can certainly borrow the paradox of the old Greek philosopher Socrates.

Indeed, molecular biology and automation are increasing the resolution capacity of our investigations into mechanisms of biological processes, shedding light on their extreme complexity.

To date, we can draw the network of relationships between, for example, bacteria and host or between tumor and surrounding normal tissue. However, we still do not understand the hierarchical sequence of events based on chronology before/after or on the cause/effect relationship between studied events.

**Figure 1:**
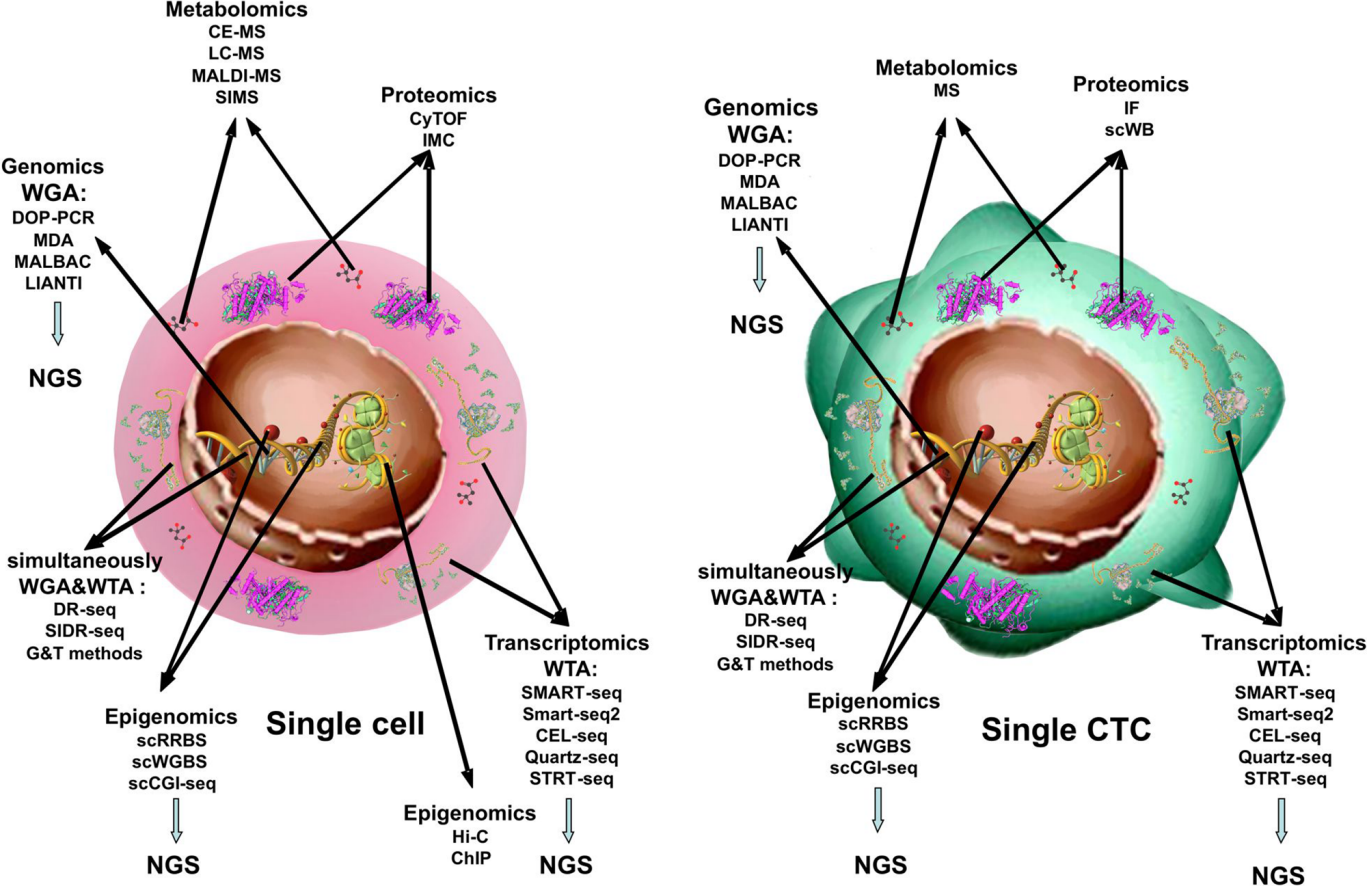
Single-cell -omics technologies. Methods used to obtain combined information about the transcriptome, genome, proteome, and epigenome from single cell and from single CTC.

Metaphorically, in cancer research, it is becoming clearer that tumor growth and progression are matters of “a criminal conspiracy” rather than of “a serial killer.” In other words, the complexity of cancer derives from the spatial and temporal heterogeneity of tumor cells, which compose the tumor tissue, and from the interplay that each of these establishes with the normal surrounding tissue. This translates into plasticity of the tumor under stress, intended as physical and chemical parameters of primary site microenvironment modified by tumor growth or by therapies, as well as interplay of tumor cells with immune or stromal cells. If we hope to manipulate tumor cells to benefit patients, we need to understand the role that each of them is playing in the overall scenario. Examining tumor cells at the single-cell level allows dissection of tumor complexity (**Figure 1**).

However, scientific knowledge on this topic is not yet complete. Studies reporting new technologies are being published almost monthly; they show that these new methods are actionable in a small pilot group of patients but often lack in robust comparison between the gold standard and new technologies. To move from benchtop to bedside, each new technology must undergo a rigorous evaluation process that includes analytical validation, clinical validation, and the demonstration of its clinical utility (Parkinson et al., 2012).

Hence, larger studies are needed before we can define clinical scenario(s) in which CTC -omics can contribute to personalized oncology. Likewise, it is not time yet to compare different scenarios of tumor evolution based on findings obtained from CTC -omics, and this review does not aim to do so.

Rather, we feel a strong need to recap the state of the art regarding the strategies to address single-cell analyses, and the respective validation levels, since the value of any finding on CTCs depends on the robustness of the method used to identify them in peripheral blood (Parkinson et al., 2012).

To this purpose, in our literature searches, we used the keyword “CTCs” and “single-cell” and “-omics”; while the keywords “single-cell” and “-omics” served to select the references on the non-neoplastic cells. We then regrouped the studies according to the main methodological challenges of the topic (enrichment of rare cells, amplification of nucleic acids) or subject of analysis (genomics, epigenomics, proteomics, multi-omics, metabolomics), briefly discussing the pros and cons of each one. We then discussed in a separate section the few published examples of clinical applications of these techniques.

Moreover, for the ideal road map of future investigations, we included two more sections: the analysis of interplay between tumor and stromal cells and the revision of clinical trial design required by new -omics approaches.

Indeed, we think that the analysis of the tumor microenvironment is fundamental in a comprehensive study of tumor evolution, and it should proceed in parallel with CTC analyses, while a critical remark on changes of validation trial schema seems mandatory, considering the depth of –omics analyses.

### What Technologies Have Been Used to Enrich and Isolate CTCs for Downstream -Omics Analyses?

#### One Cell at a Time

To date, many authors describe and summarize methods to enrich and detect CTCs. We could divide the methods to enrich and count CTCs into two groups based on the strategies used: the first-take advantage of CTC surface markers and the second uses the physical characteristics of CTCs (Amadori et al., 2009; Alix-Panabieres and Pantel, 2014; Thiele et al., 2017; Gwak et al., 2018).

A multiple -omics analysis of single CTCs would allow us to detect the spectrum of somatic mutations and changes of gene expression in patients at all stages of cancer with minimally invasive methods. In the blood stream, CTCs are exposed to several stress factors such as immune system attacks, high oxygen levels, high pressure, and cancer therapies (Alix-Panabieres and Pantel, 2014; Sarioglu et al., 2015; Wang et al., 2018), all of which could impact the quantity and quality of recovered nuclei acids.

The vast majority of researchers have analyzed bulks of CTCs for whole-genome sequencing (WGS) or whole-exome sequencing (WES) to solve problems such as cell loss or damaged genetic material to obtain high-quality sequencing libraries. However, if we are to focus on single-cell multiple-omics analysis, the majority of CTC enrichment platforms need one further step to isolate a pure single CTC. The main methods are reported below.

##### A) Laser Capture Microdissection (LCM) and Micromanipulation

These methods allow visualization of cells using a microscope, and the verification that the target cell follows relative guidelines. With micromanipulation, the operator guides a micropipette to suction the individual cells and to place them in a tube or a specific reservoir (Hu et al., 2016; Nelep and Eberhardt, 2018).

Even if this method is appropriate for the collection of a small number of cells, and previous studies have demonstrated the capability and the clinical value of sequencing single CTCs using this approach (Lohr et al., 2014; Dago et al., 2014), they are too time consuming and laborious for routine clinical applications.

LCM has been widely used to isolate single cells from solid tissue; similar to previous methods, LCM also allows an accurate target cell recovery. The sample preparation for the isolation of single rare cells causes a loss of cells, which could be high (Dhar et al., 2016). Because of this, LCM is often coupled with pre-enrichment that takes advantage of microfiltration or microfluidic devices (El-Heliebi et al., 2013; Zhao et al., 2013). Furthermore, both these strategies call for a step of cell fixation that impacts the efficacy of genotyping due to cross-links of DNA and/or whole-genome amplification failure (Zhao et al., 2013; Srinivasan et al., 2002; Carpenter et al., 2014).

To reduce cell loss and fixation problems, Park and colleagues developed an efficient workflow, which allows the isolation and sequencing of a single CTC by means of a fixation-free staining and encapsulation of the cell into hydrogel (Park et al., 2018). In this study, this workflow was applied to analyze with an NGS panel both cell lines that spiked in the blood and CTCs plus leucocytes from a prostate cancer patient. The results obtained demonstrated the feasibility of these applications.

##### B) Punch VyCAp

This method combines a slide containing a silicon chip with microwells with fluorescence imaging to identify target cells and a punching method to isolate single cells. A step of immune-depletion is necessary to reduce the leukocytes. The isolation success rate is reported to be around 80–90% (Swennenhuis et al., 2015; Stevens et al., 2018).

##### C) DEPArray System

This method combines microfluidics and microelectronics to isolate CTCs of different sizes and with different cellular surface expression properties. This system uses a dielectrophoresis-based procedure to move and capture target cells (Di Trapani et al., 2018).

Several authors combined CELLSEARCH^®^ and DEPArray^™^ to investigate not only the genetic molecular profile of CTCs but also the characteristics of single cells coming from primary tumors and metastasis (De Luca et al., 2016; Paolillo et al., 2017; Rapp et al., 2017; Lee et al., 2018). The previous version of DEPArray system was time consuming, but the DEPArray NxT has overcome this disadvantage.

#### Enough Nucleic Acids to Analyze

A single cell provides a minute amount of DNA (6 pg) and RNA (10 pg) but, to produce high-quality sequencing libraries, we need to use a larger quantity of DNA and RNA. For this reason, over the last 10 years, several methods of whole-genome amplification (WGA) and/or whole-transcriptome amplification (WTA) have been developed to amplify DNA or RNA from a single cell.

##### A) Whole-Genome Amplification

Currently, in order to amplify the genome, three methods are used: degenerate oligonucleotide-primed polymerase chain reaction (DOP-PCR), multiple-displacement amplification (MDA), and multiple annealing and looping-based amplification cycles (MALBAC). All these approaches have both advantages and disadvantages. DOP-PCR amplifies single cell–derived DNA but with a low coverage of the genome (around 10%), which is not sufficient for a good single-nucleotide variant detection (SNV); conversely, this method is useful for copy number assessment (Navin, 2014). MDA provides a non-uniform coverage of the genome and, for this reason, it is not suitable for copy number analysis but works better for the detection of mutations. Finally, with the MALBAC approach, it is possible to obtain a high and uniform coverage of the genome (93%), which is useful for the study of copy number variation but, due to a high false-positive rate, it is not suitable for the detection of point mutations (Liang et al., 2014).

In order to reduce such biases and errors, Chen and colleagues (Chen et al., 2017) have recently developed a WGA method called LIANTI, where DNA is fragmented, tagged with T7 promoter, and amplified into thousands of RNA copies. This is then followed by a step of reverse transcription and second strand synthesis into double-stranded LIANTI amplicons, which are ready to be used for library preparation. Using this method, the authors demonstrated a 97% genome coverage, a reduction in allele dropout (ADO) (<17%), and a decrease in the false-negative rate (<47%).

##### B) Whole Transcriptome Amplification

To amplify all full-length transcripts or their 3’ regions, several methods have been developed, such as SMART-seq (Picelli et al., 2013), its improved second version Smart-seq2, CEL-seq (Hashimshony et al., 2012), Quartz-seq (Sasagawa et al., 2013), and STRT-seq (Islam et al., 2011).

To create scRNA-seq libraries, Smart-seq and Smart-seq2 take advantage of a special reverse transcriptase, which anchors to both ends of cDNA with distinct nucleotides to increase the number of full-length RNAs in order to detect alternative splicing sites, genetic variants and/or new exons (Ellsworth et al., 2017). On the other hand, CEL-seq and STRT-seq target only one end of mRNA, 5’ and 3’, respectively (Hashimshony et al., 2012; Sasagawa et al., 2013; Islam et al., 2011). Quartz-seq was developed to quantify the heterogeneity of gene expression between cells. Unfortunately, each of these methods suffers from biases. Despite the use of the WTA protocol, it is rare to achieve full-length sequence of most transcripts from a single cell and, often, the least expressed transcripts might be lost (Marinov et al., 2014).

Ting and colleagues in 2014 (Ting et al., 2014) and Miyamoto and colleagues in 2015 (Miyamoto et al., 2015), in two different studies, demonstrated that only 45–60% of CTCs used for single-cell RNA sequencing contained RNA with sufficient integrity to be sequenced.

##### C) Parallel Single Cell of Genomes and Transcriptomes

With the above-described methods, only the genome or the transcriptome could be analyzed from the same cell. For this reason, it is not possible to associate changes in the genome with those in transcriptome.

In recent years, three methods have been developed to simultaneously sequence the genome and transcriptome of the same single cell. The first method, genome and transcriptome sequencing (G&T-seq), takes advantage of the physical separation of DNA and RNA immediately after cell lysis and applying Smart-seq2 and various WGA methods (Macaulay et al., 2016).

The second method, DNA and RNA sequencing (DR-seq), after cell lysis and without separating the two nucleic acids, become amplified; to amplify transcriptome, CEL-seq methods are used, and for WGA, a modified MALBAC method is used (Dey et al., 2015).

The third is simultaneous isolation and parallel sequencing of genomic DNA and total RNA from single cell (SIDR-seq); this method physically isolates nucleic acids using magnetic microbeads and total RNA from single-cell lysate, which contains the nucleus (Han et al., 2018).

For superamplification of nucleic acids, researchers combined a commercial WGA method (Repli-g Single-Cell KIT, a MDA-based WGA method), and for RNA as WTA protocol, they used SMART-seq2. In the first study, SIDR-seq appears to have some advantages over the first two methods; in fact, it is applicable to profiling of non-polyadenylated RNA (i.e., non-coding RNAs), unlike G&T methods, and seems to improve the number of reads compared to DR-seq.

However, these approaches suffer from the same biases of the WTA and WGA mentioned above.

#### One Cell, Several Study Subjects

##### A) Single-Cell Epigenomic Sequencing

With the word “epigenome,” we refer to all genomic changes which do not directly cause an alteration to primary DNA sequences but nonetheless could be hereditary. They include DNA methylation, histone modification, and chromatin binding of structural and regulatory proteins. Epigenomic changes have an impact on several aspects of gene regulation. In cancer, the epigenetic changes impact gene expression regulation; indeed, several studies highlight that an altered level of methylation (low or high) affects specific regions such as the promoter of tumor suppressor genes (Easwaran et al., 2014; Tsoucas and Yuan, 2017).

Recently, single-cell DNA methylation profiling has been developed, in particular single cell–reduced representation bisulfite sequencing (scRRBS), which is a modified bisulfite sequencing, and single-cell genome wide BS (scWGBS). Both these methods are able to analyze CpG methylome at the single-cell level but have some disadvantages; in particular, scWGBS has a higher coverage (about 81%) but with a high cost and limited consistency (21%). By consistency, we mean the intersection of all CGIs covered across a single cell. Instead, scRRBS reduced the cost but consistency is lower (1.13%) (Guo et al., 2013; Farlik et al., 2015).

Another approach, an MRE-based scCGI-seq, could improve consistency when MRE digestion is combined with MDA in order to amplify preferentially methylated CGI containing long DNA strands compared to short unmethylated CGI DNA strands and thus applied a massive sequencing. This last method has coverage similar to that of scRRBS (Han et al., 2017).

To investigate other epigenetic alterations at single-cell level, two methods have been developed: the first, Hi-C, allows the identification of trans-regulatory elements and their target, taking advantage of the capture of specific chromosome conformations (Nagano et al., 2013); the second, ChIP-seq allows the investigation of the interaction between DNA and DNA-binding proteins (Rotem et al., 2015).

##### B) Proteomic

Proteomics studies protein structures and their localization, functional status, and interactions. The transcription and translation of a gene could produce more than one protein, and these proteins could be post-translationally modified or their concentration could be temporarily modified based on their function. In cancer, kinases and phosphatases are usually deregulated and/or constitutively activated; therefore, several target drugs have been developed to contrast this disease.

Despite the fact that cells have the same genomic sequences, in most cellular systems, a huge number of subpopulations exist, which differ for phenotypes and/or functions, and a classic example is represented by PMBCs. For this reason, studding the proteome at the single-cell level could be a way to better understand heterogeneity in normal and cancer cells.

Regarding single-cell proteomics, recent progress in mass spectrometry (MS) instrumentation and sample handling strategies is quickly making comprehensive proteomic analyses of single cells feasible.

Cytometry through inductively coupled plasma time-of-flight MS (mass cytometry, also called, CyTOF, Fluidigm Corporation, South San Francisco, CA, USA) is a single-cell method which enables highly multiplexed and quantitative measurements of proteins and their modifications (Spitzer and Nolan, 2016). In this approach, the solid cancer tissue has to be split up in a viable single-cell suspension; this procedure could cause a loss of phenotypical markers or a drop in cell subset. In addition, access to fresh tissues could be a problem. However, this could be overcome using imaging mass spectrometry (IMC) (Chang et al., 2017), which also allows the detection of the entire spectrum of proteins in formalin-fixed, paraffin-embedded tissue (FFPE) in both retrospective and prospective studies.

After cell isolation, the proteomic analysis in rare subpopulations or rare cells involves several steps such as lysis, protein extraction, and digestion. Most methods require a minimum of 500–1,000 cells to analyze and identify a few hundred proteins. In 2015, Siyang Li and colleagues set up an integrated platform to profile the proteome in rare cells in the blood (Li et al., 2015), and they managed to identify 4,000 proteins in 100–200 cells.

Herr and colleagues developed a single-cell resolution western blot (scWB) that takes advantage of a microfluidic tool for the isolation of single cell, and a microscope slide coated with a photactive polyacrylamide gel, aligned with an open microwell array where single cells are lysed (Kang et al., 2016). This western blotting approach allows the profiling of protein expression in single rare cells including CTCs (Sinkala et al., 2017).

##### C) Metabolomic

To profile the metabolome in cancer, metabolomics approaches could allow us to understand the deregulation of metabolism and metabolic vulnerabilities in cancer. The metabolic profile often reflects alterations in the genome, epigenome, and proteome. Recent, studies demonstrated that metabolites could initiate cellular signaling cascades and modulate biological processes such as epigenetic mechanisms and post-translational modifications (Johnson et al., 2016; Duncan et al., 2018).

For this reason, metabolomics research is being used to discover diagnostic cancer biomarkers for clinical use; therefore, to discover pathways involved in cancer, it is essential to better understand the complex heterogeneous nature of the metabolome, which could result in new target drugs and methods to monitor metabolic biomarkers during treatment. Single-cell metabolomics offers many opportunities, but also many challenges due to the limited sample volume, low analyte amounts, and rapid turnover rates of cellular metabolites.

To study metabolites, NMR and MS are the main analytical approaches which typically require a large number of cells for sample preparation (Wang et al., 2010). Developing analytical tools for single-cell metabolomics is challenging, because, in metabolome analysis, no amplification strategies exist (Comi et al., 2017). Many MS-based techniques have been extensively used in single-cell analysis, such as capillary electrophoresis–mass spectrometry (CE-MS), (Aerts et al., 2014) liquid chromatography–mass spectrometry (LC-MS), matrix-assisted laser desorption/ionization–mass spectrometry (MALDI-MS) (Ong et al., 2015; Ibanez et al., 2013), and secondary ion mass spectrometry (SIMS). All these approaches have been largely applied in both microorganisms and plants. With regards to cancer, most studies analyzed cell lines and were rarely applied to cancer cells or to circulating cancer cells. However, these studies represent the proof of concept that single-cell metabolomic analyses are possible and could soon be applied not only to cell lines or cells derived from model organisms but also to patient samples (Duncan et al., 2018).

Some challenges still lie ahead as new metabolite, and metabolic pathways are continuously being discovered. Therefore, it is necessary to accurately annotate the detected m/z value, to improve the free metabolic database developed for MS and MS/MS data and to develop specific bioinformatic tools for single-cell metabolome analysis.

#### Bioinformatics

Traditional -omics studies measured the level of thousands of analytes in thousands of cells, while single-cell data sets show a high level of missing information due to the diluted analysis. Simultaneously, new laboratory platforms and methods aim to improve -omic analyses and the amount of data obtained from a single cell, whereas bioinformaticians, engineers, and statisticians develop methods and tools to analyze all these data sets to reveal new insights to be applied in personalized medicine.

Over the past few years, many methods have been designed to analyze single-cell DNA-seq data. When we apply these methods to CTC data, we have to set up bioinformatic analyses considering the biases inserted during sample preparation and library creation (Zhu et al., 2018; Castro-Giner et al., 2018).

To date, Monovar is the only variant caller tool specifically developed to analyze single-cell data. It detects any SNV taking into consideration all biases inserted by WGA protocols (Zafar et al., 2016; Ramalingam and Jeffrey, 2018).

To study cancer evolution using sc-sequencing data, two tools are available: OncoNEM and SCITE. These methods allow the identification of mutations that occur during tumor development from the early to late stages, which could help to understand the development of therapy resistance. SCITE uses the Markov chain–based Monte Carlo algorithm, and OncoNEM uses a heuristic search. These tools build sequencing error estimation using maximum likelihood principles to overcome sc-sequencing biases (Tsoucas and Yuan, 2017).

To analyze scRNA-seq data, two approaches are often used: differential expression analysis and clustering analysis. In general, single-cell data have to be normalized, and several tools have been developed for specific single-cell data analysis, for example, SCDE, MAST, and Basic.

To address the challenge of data integration, two approaches have been used, Sc-map and Seurat, which focus on the dimensional reduction to internally normalize different datasets uncovering the underlying, low-dimensional manifolds, which are conserved in biological samples (Leonavicius et al., 2018).

Recently, in order to study the regulatory networks of specific genes, Chiu et al. developed scdNet, which features two functions: gene correlation analysis out of sparse data matrices and differential network analysis between cellular states (Chiu et al., 2018).

To identify rare cell types from scRNA-seq data, three clustering methods have been developed: RaceID, its update RaceID2, and GiniClust. With RaceID/RaceID2, cells are clustered into major groups (by K-means in RaceID or K-medoid clustering in RaceID2) and clusters of outliers. After the elimination of clusters due to technical or biological noise, they are then grouped into rare clusters based on transcriptome correlation (Tsoucas and Yuan, 2017; Grun et al., 2015; Grun et al., 2016). On the other hand, GiniClust uses the Gini index to choose genes that are likely to be associated with rare cell types (Tsoucas and Yuan, 2017; Jiang et al., 2016).

Matcher is a tool which allows the use of different -omic data sets, e.g., epigenome and transcriptome, to create an equivalent pseudo-time representation between these data sets, useful for alignment (Welch et al., 2017).

Regarding single-cell proteomic data, specific tools such as Spada, PhenoGraph, and Wishbone are available to infer subpopulation markers. HistoCAT, another statistical tool, is used to detect spatial and phenotypic interactions at the cellular level, integrating single-cell CyTOF measurements and image-based spatial information (Ortega et al., 2017).

Finally, the development of specific bioinformatic tools for single-cell metabolomic analysis is just starting (Duncan et al., 2018).

### How Far Have We Come in the CTC -Omics Era?

Morphological heterogeneity is a well-known property of tumor tissues that pathologists have classified into grading schemes, including nucleus shape, invasion level of surrounding normal tissues or micro-vessels, and necrotic fraction of the sample. Depending on the histology of the tumor, these characteristics often have a prognostic value, and the introduction of immunohistochemistry in routine analyses has further consolidated this concept, helping to choose the most suitable treatment for each patient profile. The introduction of high-throughput technologies now allows distinguishing different sub-clones in the tumor, longitudinally monitoring their evolution. Above all, the resolution at the single-cell level promises to answer the main question, namely, if tumor heterogeneity increases or decreases during disease progression and what is the evolutive pressure exerted by anti-cancer treatments.

To address tumor heterogeneity, by combining flow sorting and gene expression microarrays, Naik and coll. (Naik et al., 2016) profiled, in a xenograft model of ovarian cancer, the molecular landscape of different cell types, based on their regenerative hierarchy and genetic instability, and identified their relevance to tumor behavior.

Strikingly, these technologies can also be applied to CTCs. For example, single-cell RNA-seq is already being used to investigate origin, phenotype, and drug-resistance pathways of CTCs (Ting et al., 2014; Miyamoto et al., 2015).

Despite the very low number of CTCs and their high level of heterogeneity, there are an increasing number of studies that exploit CTCs to trace cancer progression and the development of therapy resistance. By understanding tumor heterogeneity and tumor evolution, the pragmatic goal is to find new therapeutic options. Conversely, the use of CTC -omics as a diagnostic tool is not affordable, since the clinical demonstration of patient benefit, such as for quality of life or longer survival, at a lower cost for Public Health System is lacking (Parkinson et al., 2012).

De Luca et al. showed, in a breast cancer patient, how the majority of CTC mutations detected at baseline disappeared during treatment and new mutations emerged (De Luca et al., 2016).

The whole CTC genome sequencing in prostate cancer, in a study by Dago et al., allowed the discovery of two distinct CTC subpopulations with an amplified androgen receptor (AR) gene. Interestingly, they demonstrated that one of these subpopulations descends from a cancer clone found in therapy-naïve patients (Dago et al., 2014). In the same paper, the authors observed that, in CTCs, CNV evolution reflects clinical response and/or disease progression. One year later, Miyamoto et al. applied scRNA-seq to CTCs in 13 drug-resistant prostate cancer patients and demonstrated AR-independent resistance acquired with the non-canonical Wnt signaling pathway (Miyamoto et al., 2015).

Aceto and colleagues, in women with ER+ breast cancer, applied RNA-seq on isolated CTCs. They found in CTCs from bone-predominant breast cancer an AR signaling pathway constitutively activated due to a splicing variant named AR-v7. The authors highlighted the role of the AR in breast cancer bone metastasis and suggested that metastatic breast cancer patients may benefit from an AR target therapy (Aceto et al., 2018).

In hepatocellular carcinoma (HCC), D’Avola and colleagues applied scRNA-seq in enriched CTCs without applying WTA but took advantage of the Chromium platform (Chromium 10X) to create libraries. The authors underlined how CTCs expressing HCC upregulated the long non-coding RNA (HULC) and, most importantly, they detected the overexpression of IGF2 that could be used to design a target therapy. In HCC, the most prevalent mutations are not “druggable” and, for this reason, this CTC study suggested that oncologists could apply a target therapy against IGF2, which is not usually used in HCC patients (D'Avola et al., 2018).

The methylation analysis in metastatic breast cancer CTCs, which are isolated from 17 patients, showed different subpopulations of CTCs that could be identified with methylation profiling (Benezeder et al., 2017). The authors further highlighted that patients with methylated CTCs for genes CST6, ITIH5, and RASSF1 had a significantly shorter PFS compared to patients with non-methylated CTCs (Benezeder et al., 2017).

Regarding past CTC protein studies using immunostaining and/or immunofluorescence, several authors highlighted that CTCs could change their phenotype during treatment (de Bono et al., 2007; Rossi et al., 2012; Krebs et al., 2012; Lindsay et al., 2016; Jaeger et al., 2017).

With the rare-cell scWB, in 2016, Sinkala et al. identified differences in CTC protein expression profiles, including both wider EpCAM expression range and subpopulations with distinct GAPDH expression levels (Sinkala et al., 2017).

A single-cell metabolomic study demonstrated that CTCs from gastric cancer (GC) and colorectal cancer (CRC) have a different lipid profile. In fact, in CRC, CTC sterol lipids (SL), and acyl carnitine are high. On the contrary, in GC CTCs, authors detected a high level of fatty acids (FA) and glycerophospholipids (GPLs). Previous studies demonstrated a link between high levels of SL and distant metastases in CRC, and how GPL synthesis is involved in cancer proliferation, specifically in the membrane, and in energy production in GC (Abouleila et al., 2018).

In order to obtain a greater number of CTCs, many authors have tried to culture CTCs, or to create a xenograft from CTCs. In particular, Jordan NV et al., studying 19 ER+/HER2− breast cancer samples, detected 16 out of 19 CTC-positive patients with HER2 expression but without HER2 amplification. They expanded CTCs in culture and, using FACS, sorted the two subpopulations HER2+ CTCs and HER2-CTCs; then, they applied MS. The MS analysis highlighted that HER2+ CTCs showed an increased expression in receptor tyrosine kinase genes and HER2-CTCs an increased NOTCH signaling. They observed how the two subpopulations are able to interconvert spontaneously, and that chemotherapy causes an enhanced transition from HER2+ to HER2-CTCs. Moreover, they used CTCs derived from the tumor in mouse model to test drug susceptibility, demonstrating that a combination of paclitaxel and Notch inhibitors suppressed tumorigenesis (Jordan et al., 2016).

### Investigating Tumor Microenvironment

Single-cell genomics are allowing us to distinguish cellular from sub-cellular drivers of biological processes in complex primary samples. For example, Gierahn and coll. have profiled thousands of primary human macrophages exposed to tuberculosis, within PBMCs (Gierahn et al., 2017), finding that basal cellular heterogeneity may influence the overall response to tuberculosis. Similar, the single-cell resolution allowed Tang and coll. (Yan et al., 2013) to provide a comprehensive view of the pre-implant human embryo transcriptome, reporting first that gene expression signatures of human epiblast (EPI) dramatically differ from primary human embryonic stem cell (hESC) outgrowth.

To date, metastasis remains a poorly understood process because of the complex interplay that the primary tumor establishes with stromal cells, often based on redundant and still unclear signaling pathways (Lambert et al., 2017). Among stromal cells, those of the immune system especially affect the outcome of tumor progression and metastasis. Indeed, -omics technologies can dissect this complexity, since we can now analyze in parallel tumor cells and normal surrounding tissues, and for this reason, we briefly review the studies that addressed the interplay between CTCs and immune and non-immune stromal cells.

Abangan and coll. reported in a mouse model that hematopoietic stem cells (HSCs) are a novel source of carcinoma-associated fibroblasts, the circulating fibroblast precursors (CFPs), and demonstrated the role of monocyte chemo-attractant protein (MCP1) in regulating their contribution to the tumor microenvironment (Abangan et al., 2010).

Tirosh and coll. combined flow cytometry sorting of individual viable immune (CD45+) and non-immune (CD45−) cells, followed by classification based on preferentially or uniquely expressed marker genes. With this more complex procedure, they profiled the human metastatic melanoma at the single-cell level, including both malignant and non-malignant cells [T cells, B cells, macrophages, endothelial cells, cancer-associated fibroblasts (CAFs) and NK cells] (Tirosh et al., 2016).

Through this approach, the authors were able to reveal intra- and inter-individual, spatial, functional, and genomic heterogeneity, not only in melanoma cells but also in associated tumor components, which constitute the microenvironment, including immune cells, CAFs, and endothelial cells. Moreover, in all melanomas, they were able identify a subpopulation linked to drug resistance.

Immune cells deceptively show both anti- and pro-tumor effects (Leone et al., 2018). In head and neck squamous cell carcinoma (HNSCC), Strauss and coll. (Strauss et al., 2007) reported that Tregs, which accumulate in the tumor tissue, differ phenotypically from those among the PBMCs; moreover, they have higher suppressive capacity. Furthermore, tumor Tregs are associated with a poor prognosis in HNSCC patients.

Metastatic breast cancer patients with >5 CTCs per 7.5 ml of blood had circulating NK cells showing a deficient lytic ability in chromium-51 release assay as compared to those from patients with ≤5 CTCs, and an inverse correlation between CTCs and progression-free survival (PFS) was found (Green et al., 2013).

Moreover, in metastatic breast, colorectal, and prostate cancer patients, the cytotoxic activity of NK cells inversely correlated with the CTC levels detected by the CELLSEARCH System (CS, Menarini Silicon Biosystems) (Santos et al., 2014).

A study performed on late stage NSCLC unveiled CTCs expressing both epithelial and mesenchymal markers in RNA-ISH assays, which negatively correlated with CD3+ and CD8+ T cells. Moreover, CTC levels positively correlated with metastases and a worse clinical outcome (Sun et al., 2017).

Mego et al. also showed a correlation between CS-derived CTC counts and the percentage of activated IL-17–producing CD8+ T cells (Mego et al., 2016).

MDSCs are heterogeneous, immature myeloid cells comprising a polymorphonuclear subset (PMN-MDSCs) and a monocytic subset (M-MDSCs), which can be respectively distinguished from granulocytes and monocytes due to their high immunosuppressive activity (Ostrand-Rosenberg and Fenselau, 2018). Interestingly, in a study on portal vein blood samples from pancreatic cancer patients, the authors found a correlation between the number of circulating M-MDSCs and active FACS-isolated K-RASmut mRNA+ CTCs, suggesting that the establishment of liver metastases in these subjects may be supported by immunosuppression-dependent CTC survival in the bloodstream (Arnoletti et al., 2017).

High-throughput technologies, such as next-generation sequencing (NGS), and computational tools for data analysis are now available to study tumor escape mechanisms and to unveil still unknown interactions between tumor and immune cells (Hackl et al., 2016).

Recently, Hong and coll. isolated CMCs from 16 metastatic melanoma patients undergoing therapy with ipilimumab, by means of microfluidic enrichment, and developed a CTC scoring assay to evaluate a 19-gene digital RNA signature. They showed that the use of this quantitative CTC score, applied to the serial monitoring of patients, was predictive of long-term response to immunotherapy. This offered an alternative to the analysis of repeated tumor biopsies, which are invasive and insufficiently precise to guide new or ongoing treatments (Hong et al., 2018).

In the near future, we will be able combine several -omics technologies (including genome, epigenome, transcriptome, proteome, and metabolome) into integrated assays on the same single cell. Thanks to the bioinformatic analyses, we will yield a single multi-omics map, providing a data-driven model of the complex tissue and cellular lineage hierarchies. Hopefully, we will be able to move a step forward and use this knowledge for the benefit of cancer patients (Bock et al., 2016).

### New Designs of Clinical Trials

New technologies allow more in-depth profiling of the molecular landscape of individual tumors and, since there is an increasing number of drugs specific for any molecular aberration, new schemes for clinical trials are being introduced devoted to the assessment of the activity of any targeted drug in specific molecular subgroups of cancer (Zardavas and Piccart-Gebhart, 2015).

Undoubtedly, the high inter-tumor heterogeneity represents the main challenge of validation of targeted drugs, because most aberrations are reported, for example, in less than 10% of analyzed breast cancer (Prat et al., 2010; Gucalp and Traina, 2010). This condition has increased the number of patients to be recruited and requires functional validation of various aberrations of each pathway.

**Master-protocol trials** can address these difficulties. Indeed, this kind of study simultaneously investigates different targeted drugs within molecular sub-groups of patients. Because of the collaborative design of master-protocol trials, screening failures (and related costs) can be reduced by enrolling patients with different aberrations in one of the different molecularly defined cohorts.

Another example of an entirely molecularly driven study design is the **basket trial** that recruits patients based on a specific molecular aberration, regardless of histology. Even in this case, the assessment of treatment efficacy in patients carrying molecular aberrations (not yet reported in that malignancy or relatively rare) might be speeded up by findings obtained in malignancies of different histology. However, we cannot exclude that the same mutation might determine different effects in different tissues (Corcoran et al., 2012).

Furthermore, the extensive molecular investigations of malignancies unveiled high levels of intra-tumor heterogeneity. As a consequence, it is becoming clear that, to be effective, precision medicine would require tailoring treatment strategy to each individual tumor. In addition to the clonal evolution that cancer undergoes during the natural history of the disease and under treatment pressure, spatial and temporal heterogeneity represents one of the main challenges to carry out truly tailored treatment and to demonstrate its clinical utility.

To speed up clinical research on this new frontier, the strategy of **N-of-1 trial** has recently received great attention as a way to overcome the conventional study design of a one-size-fıts-all paradigm. The N-of-1 trial refers to a specific design that uses repeated cycles of treatments in a single patient, in which the first is the test drug, and the second is the comparison drug. According to this scheme, one patient serves as her/his own control.

The N-of-1 trial has not been used frequently in oncology and has required some modifications to generalize results (Collette, 2015). The first thing to be careful of is that we need many cycles of repeated treatments to exclude intra-patient variability. Secondly, a proper washout period should be planned to avoid carry over from one treatment to another. Overall, the N-of-1 trial seems to better fit with chronic disease, when we have a reasonable time lapse of a relatively stable disease. However, it undermines some strong founding concepts of clinical oncology, namely, to interrupt an effective treatment or re-use some previously employed molecules in the same patient.

Consequently, the N-of-1 trial seems more suitable in the case of rare tumors, in off-label use of a treatment, or when re-cycling is permitted, as in the case of immunotherapy (Catani et al., 2017).

A recent systematic review (Li et al., 2016) of N-of-1 trials published between 1985 and 2013, which evaluated their consistence to reporting guidelines [CONSORT Extension for N-of-1 Trials (CENT)] (Turner et al., 2012). The authors concluded that N-of-1 trials published in close to 30 years show variable levels of quality. Because of the strongly patient-oriented nature, the better the study design, the treatment choice, and the reporting, the greater the generalizability of results will be.

In this respect, the interest of researchers is strongly catalyzed in pooling data from single patients treated with molecularly driven drugs (and immunotherapies), since these could be then queried to assess preliminary efficacy in a larger patient cohort (Catani et al., 2017). Special attention should be put on collecting liquid biopsies, and perhaps biopsies of the most accessible lesions throughout the trial, to prospectively evaluate biological mechanisms of treatment-induced resistance.

Similarly, because of the enormous spread of personal computing technologies, interest in Patient Generated Health Data (PGHD) is emerging. PGHD includes health history, symptoms, biometric data, treatment history, lifestyle choices, and other information recorded by patients or their caregivers. These data could unveil mechanisms of treatment action or predict treatment-related toxicity, knowledge useful to improve patient compliance and clinical outcomes (Wood et al., 2015).

In addition, the use of new schemes for clinical trials is opening a new front on ethical issues. In randomized clinical trials, oncologists collect up to hundreds of patients, a number that guarantees a complete statistical analysis of the effects of new treatments. By reducing the sample size, we might not be able to predict if our patient will respond to treatment or not. Similarly, the small sample size might limit knowledge about toxicities, which is not revealed in a series of small trials. The oncologist should adequately inform the patients about these issues, allowing them to provide their own free and informed consent to participate in this kind of studies. In addition, the new -omics technologies might reveal incidental findings of genetic information with potential effects on members of the patient’s family. This possibility should be adequately discussed with the patient, and the informed consent should be adapted to these novel needs (Gupta et al., 2017).

### Moving From Benchtop to Bedside

With the advent of the -omics era, we are able obtain the profile of individual patients, with all their driver mutations, and match these mutations with an increasing number of mutation-specific approved/experimental drugs. Exploiting -omics technologies at the single-cell level promises further unveiling of metastasis and resistance mechanisms; however, we should introduce some cautionary notes when moving from benchtop to bedside.

Increasing targeted drugs are available almost monthly; thus, oncologists have an urgent need for new tools to select patients who will benefit from a particular treatment, since we cannot monitor new therapies with old markers.

However, we should be pragmatic in distinguishing research from clinical purposes and define thresholds below which we cannot obtain informative results for treatment options, at least with the current technologies. Research continues, but patients should be conscious of the level of their altruistic participation to any study.

Second, even with a multidisciplinary team of oncologists, geneticists, pharmacologists, immunologists, and bioengineers as the best decisional tree, we still risk falling short of the target, if we do not consider the longitudinal evolution of malignancies (Silvestris et al., 2017). To this purpose, the introduction of liquid biopsy in its different forms (CTCs, cfDNA, tdEVs, exosomes, and so on) in clinical practice should be encouraged as companion diagnostics. Indeed, liquid biopsy seems to be the only class of biomarkers able to mirror tumor evolution over the course of disease. The cooperative effort made by international consortia, such as CANCER_ID (https://www.cancer-id.eu/), in standardization of methodologies is moving in this direction. We think that liquid biopsy data should be part of the new design of clinical trials.

Finally, in constructing new trials in oncology, we should keep in mind the biological nature of the new targeted-drugs, since this requires revising the use of the maximum tolerated dose of any agent. This dogma of clinical research is based on the linear relationship between drug dose and cellular death. This procedure is correct for chemical agents, but it is unsatisfactory in case of biological agents, especially those that depend on receptor/ligand interactions. Indeed, in this last case, maximum inhibition is reached with the dose fully inhibiting the target (U-shaped response curve) (Klement et al., 2016), and any further increase in the dose only intensifies toxicities. This recommendation is relevant, since it is conceivable that, in the future, different targeted drugs might be used in sequence or in parallel, to fight different tumor cells or stromal cells involved in the metastatic process.

## Conclusions

Despite the developments in single-cell isolation techniques, single-cell -omics, and bioinformatics, the acquisition of knowledge in the field of single-cell -omics of CTCs is slower than in other fields.

This is probably due to the rarity and frailty of CTCs that, together with the stress conditions applied to isolate single cells, determine a slowdown in CTC-omics studies.

In the near future, new microfluidic devices to isolate cells, and/or the droplet technology combined with barcoding—to recognize DNA, RNA, proteins, and metabolites belonging precisely to single CTCs in a mixture of leucocytes—could allow us to overcome this impasse. Nevertheless, to date, CTC characterization is starting to shed light on tumor heterogeneity and therapeutic resistance mechanisms.

We believe that soon we will be able to identify new pathways specific for metastasis-competent and therapy-resistant CTCs, which will lead to the development of novel drugs aimed at their eradication and the creation of a simple tool for managing patients in clinical practice.

## Author Contributions

ER and RZ critically reviewed the scientific literature discussed in this article; both authors wrote the article.

## Funding

The concepts developed in this review derived from the work performed at the CTC-laboratory of IOV-IRCCS, Padua, Italy, and partially funded by Innovative Medicine Initiative Joint Undertaking [115749] CANCER-ID; Intramural 5X1000 SINERGIA, IOV-IRCCS, “CTC/cfDNA” (PI: RZ); Intramural 5X1000 SINERGIA, IOV-IRCCS, “NSCLC: Mutational and metabolic characteristics of Circulating Tumor Cells (CTC)” (PI: ER).

## Conflict of Interest

The authors declare that the research was conducted in the absence of any commercial or financial relationships that could be construed as a potential conflict of interest.

The handling Editor declared a shared affiliation, though no other collaboration, with one of the authors ER.

## Abbreviations

CTCs, circulating tumor cells; DOP-PCR, degenerate oligonucleotide-primed polymerase chain reaction; MDA, multiple-displacement amplification; MALBAC, multiple annealing and looping-based amplification cycles; LIANTI, linear amplification *via* transposon insertion; G&T-seq, genome and transcriptome sequencing; DR-seq, DNA and RNA sequencing; SIDR-seq, simultaneous isolation and parallel sequencing of genomic DNA and total RNA; scRRBS, single-cell reduced representation bisulfite sequencing; scWGBS, single-cell genome wide bisulfite sequencing; scCGI-seq, single-cell genome-wide CpG island methylation sequencing; Hi-C, high-throughput chromatin interactions; ChIP-seq, chromatin immunoprecipitation sequencing; MS, mass spectrometry; CyTOF, cytometry through inductively coupled plasma time-of-flight mass spectrometry; IMC, imaging mass spectrometry; scWB, single-cell resolution western blot; IF, immunofluorescence; NMR, nuclear magnetic resonance; CE-MS, capillary electrophoresis–mass spectrometry; LC-MS, liquid chromatography–mass spectrometry; SIMS, secondary ion mass spectrometry; NGS, next-generation sequencing.
